# Fenugreek supplementation during high-fat feeding improves specific markers of metabolic health

**DOI:** 10.1038/s41598-017-12846-x

**Published:** 2017-10-06

**Authors:** Eric J. Knott, Allison J. Richard, Randall L. Mynatt, David Ribnicky, Jacqueline M. Stephens, Annadora Bruce-Keller

**Affiliations:** 1 Pennington Biomedical Research Center, Louisiana State University System, Baton Rouge, LA 70808 USA; 2 Department of Plant Biology, Rutgers University, New Brunswick, NJ 08901 USA

## Abstract

To assess the metabolically beneficial effects of fenugreek (*Trigonella foenum-graecum*), C57BL/6J mice were fed a low- or high-fat diet for 16 weeks with or without 2% (w/w) fenugreek supplementation. Body weight, body composition, energy expenditure, food intake, and insulin/glucose tolerance were measured regularly, and tissues were collected for histological and biochemical analysis after 16 weeks of diet exposure. Fenugreek did not alter body weight, fat mass, or food intake in either group, but did transiently improve glucose tolerance in high fat-fed mice. Fenugreek also significantly improved high-density lipoprotein to low-density lipoprotein ratios in high fat-fed mice without affecting circulating total cholesterol, triglycerides, or glycerol levels. Fenugreek decreased hepatic expression of fatty acid-binding protein 4 and increased subcutaneous inguinal adipose tissue expression of adiponectin, but did not prevent hepatic steatosis. Notably, fenugreek was not as effective at improving glucose tolerance as was four days of voluntary wheel running. Overall, our results demonstrate that fenugreek promotes metabolic resiliency via significant and selected effects on glucose regulation, hyperlipidemia, and adipose pathology; but may not be as effective as behavioral modifications at preventing the adverse metabolic consequences of a high fat diet.

## Introduction

Plants have a long history of medicinal applications across cultures, and many modern pharmaceuticals were originally derived from botanicals. Fenugreek (*Trigonella foenum graecum* L.) (FG) is a plant whose seeds have been historically used in cooking and as an herbal remedy, with anti-microbial, anti-inflammatory, anti-oxidant, anti-cancer, and anti-diabetic effects [reviewed in^1^]. Several studies in animal models of pharmacologically-induced diabetes^2–8^ support its efficacy as an anti-hyperglycemic agent^2–23^. In these studies, the anti-diabetic effects of fenugreek have been attributed to several biological mechanisms, including inhibition of intestinal glucose absorption^2–4^, delayed gastric emptying^3^, and insulinotropic activity^5–8^. Other evidence supports a role for fenugreek in protecting against hepatic steatosis^14–18^, inflammation^18–21^, or the oxidative stress secondary to diabetes^9–13^. Notably, hypoglycemic and hypocholesterolemic effects of fenugreek have been observed in mice, rats, dogs, rabbits, and humans [reviewed in^24^]. Despite the large number of studies performed, results have been inconsistent, possibly due to differences in study design, disparate model systems, and different methods used for botanical delivery (in food vs. gavage). In a clinical trial, fenugreek seed powder improved glucose tolerance by more than 20% after a two-week treatment of non-diabetics^25^. In another trial, 24 diabetic patients were treated with water-soaked fenugreek seeds for 8 weeks and significant reductions in fasting blood glucose, triglycerides and VLDL-cholesterol were observed^26^.

To examine the metabolic effects of fenugreek, we administered high-fat (HF, 60% energy) or nutritionally matched low-fat (LF, 10% energy) diets supplemented with fenugreek (2% w/w) to male C57BL/6J mice, a widely-used animal model of human obesity, insulin resistance, and Type 2 diabetes^27^. To thoroughly document the metabolic effects of fenugreek, we regularly assessed body weight, adiposity, glucose and insulin tolerance, and documented energy expenditure. We also examined insulin secretion and lipid accumulation in liver as well as adiponectin expression in subcutaneous adipose tissue. To date, there are only two other studies by one research group that have used the highly studied C57BL/6J mouse model to examine anti-diabetic effects of fenugreek^28,29^. These studies relied on fasting glucose/insulin levels and HOMA-IR as a proxy for insulin resistance. Although many of our observations are consistent with these studies, we incorporated powdered fenugreek seed into the diet rather than performing oral gavage with ethanolic extracts. In addition, we performed a more comprehensive analysis and compared the effects of voluntary exercise (wheel running, WR) to fenugreek supplementation in mice fed a high-fat diet. Although both fenugreek supplementation and wheel running both improved glucose tolerance, four days of exercise was overall more effective at lowering body weight and improving glucose tolerance.

## Results

### Fenugreek supplementation modestly improves glucose tolerance without affecting insulin tolerance, body weight, or body composition

**Figure 1:**
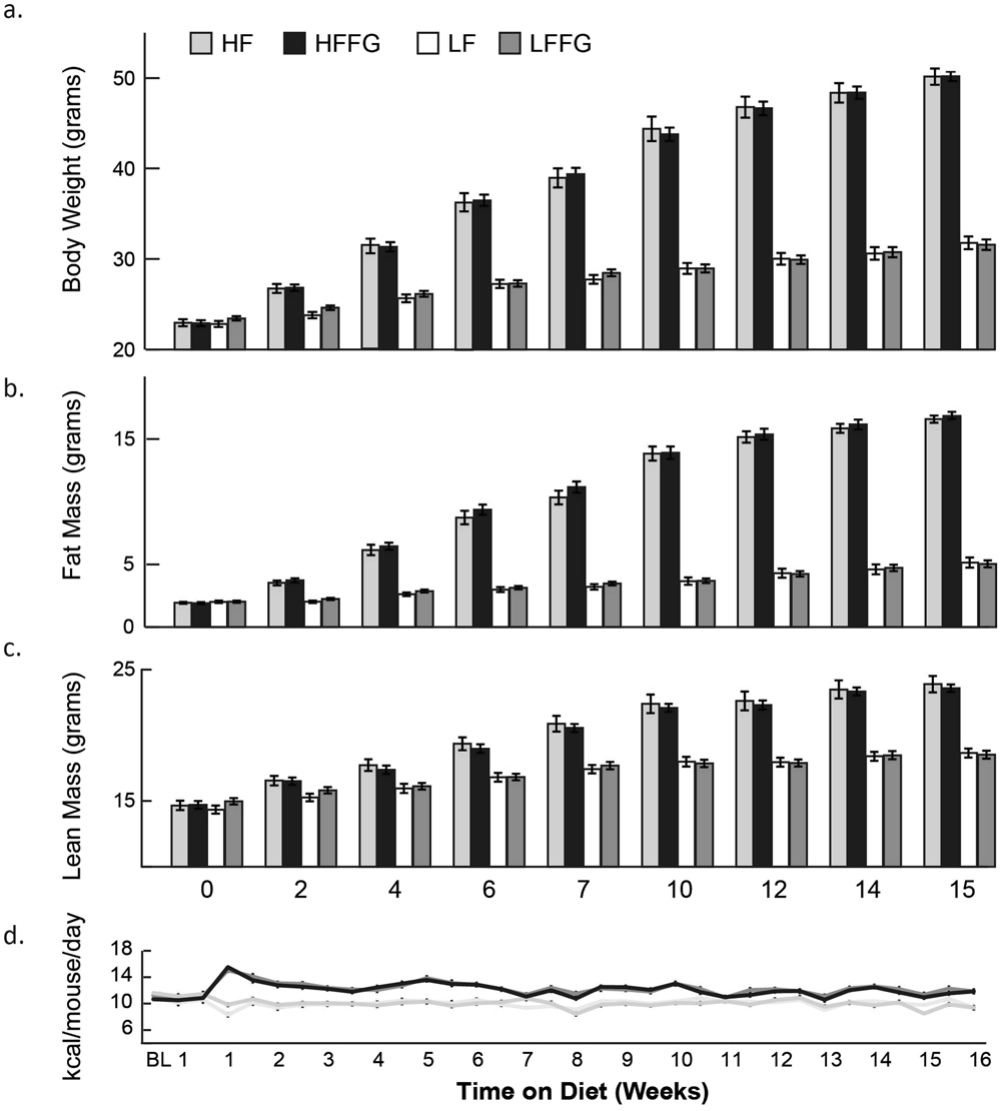
Fenugreek supplementation did not alter food intake or body weight of LF- or HF-fed mice. (**a**) Body weight, (**b**) fat mass, and (**c**) lean mass, were measured every two weeks, while (**d**) food intake was measured twice a week during 16 weeks of HF or LF-diet ± FG. Results are presented as means ± SEM (*n* = *20*). Comparisons were made by a 1-way ANOVA followed by student t-test. (*n* = *20*) *p < 0.05 was considered significant. LF, low-fat diet control; HF, high-fat diet control, LFFG, LF + 2% *Trigonella foenum-graecum* (fenugreek); HFFG, HF + 2%*Trigonella foenum-graecum* (fenugreek).

**Figure 2:**
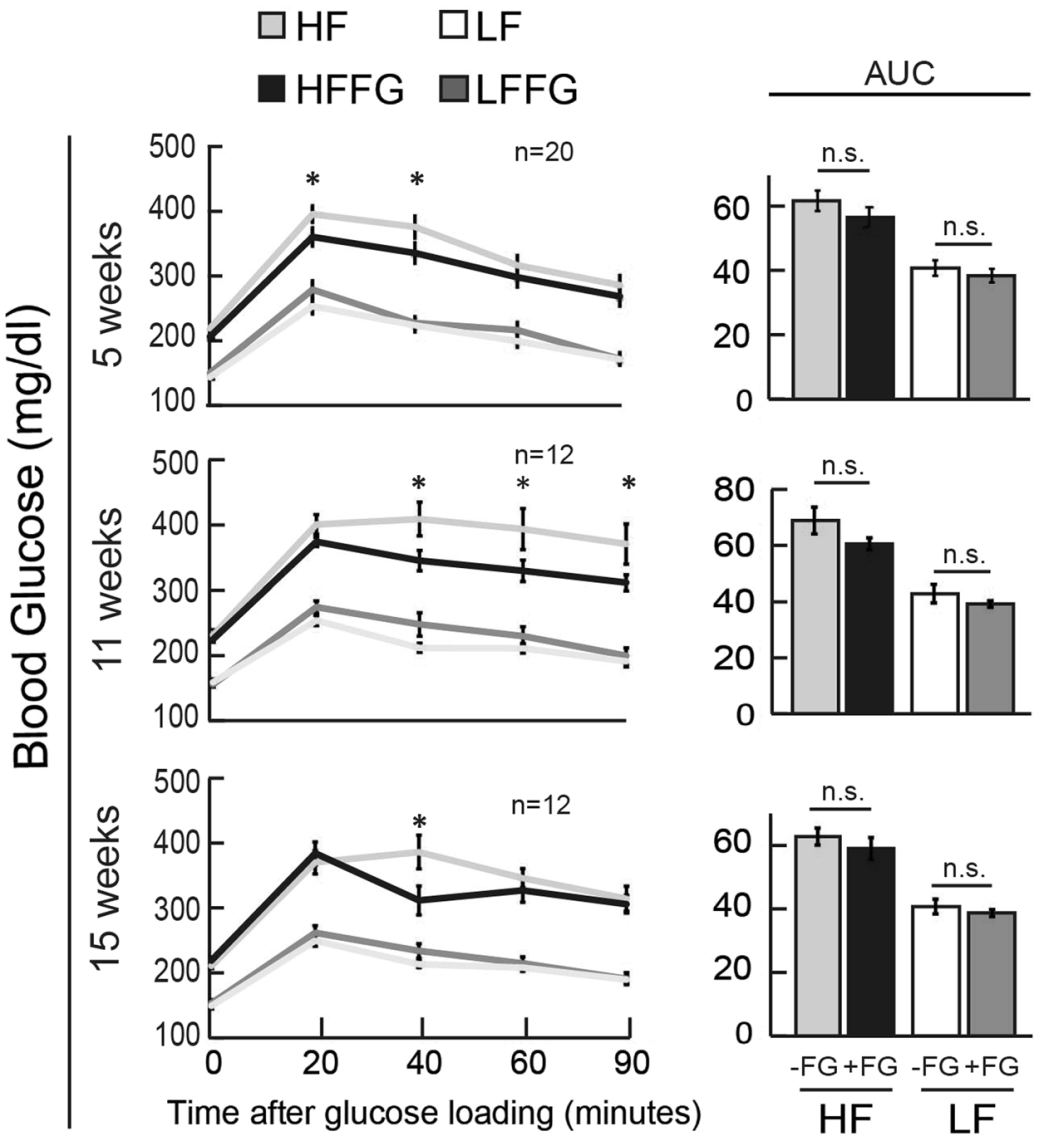
Fenugreek supplementation mildly improved serum blood glucose levels during oral glucose tolerance test in HF-fed mice. Oral glucose tolerance tests (OGTT) were performed at 5, 11, and 15 weeks on mice fed a LF, HF, LFFG or HFFG diet. Area Under the Curve (AUC) of OGTT at 5, 11, and 15 weeks. Data are presented means ± SEM (*n* = *20 &* *12*) as indicated. The AUC values were subjected to 1-way ANOVA and differences between means were obtained by student t-test. *p < 0.05 was considered significant.

**Figure 3:**
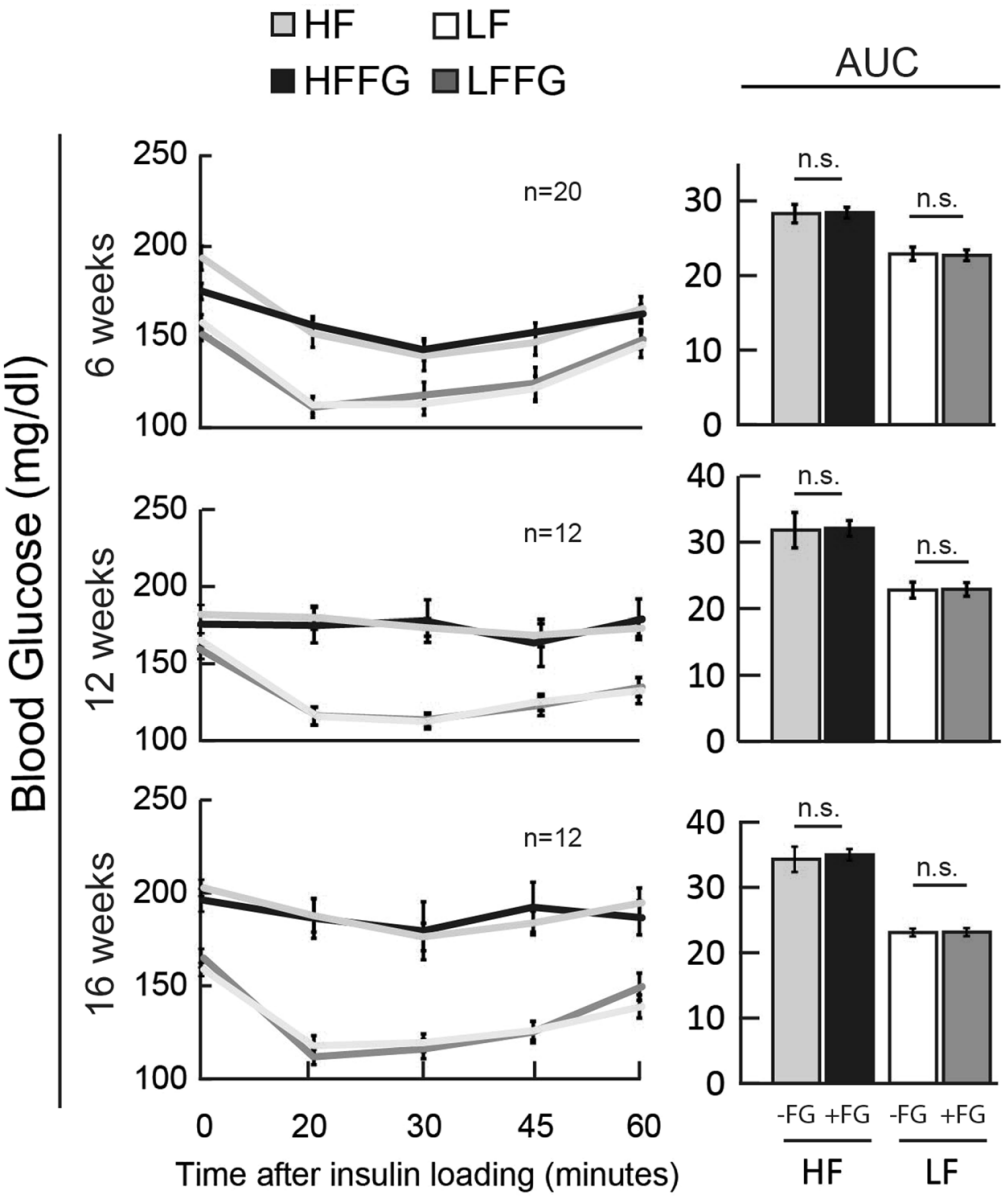
Fenugreek supplementation did not improve insulin tolerance in LF- or HF-fed mice. Insulin tolerance tests (ITT) were performed at 6, 12, and 16 weeks on mice fed a LF, HF, LFFG, or HFFG diet. AUC of ITT at 6, 12, and 16 weeks. Data are presented as means ± SEM, (*n* = *20 & 12*) as indicated. The AUC values were subjected to 1-way ANOVA and differences between means were obtained by student t-test. *p < 0.05 was considered significant.

To determine the effects of fenugreek supplementation on body weight, body composition, and whole body glucose homeostasis in the context of a high-fat diet, we fed C57BL/6J mice either a LF- or a HF-diet with or without fenugreek (2% w/w) (LFFG or HFFG) for 16 weeks. We measured body weight and composition at two-week intervals, and performed an oral glucose tolerance test (OGTT) at weeks 5, 11, and 15, as well as a insulin tolerance test (ITT) during weeks 6, 12, and 16. Comparison of LF- to LFFG mice revealed no effects of fenugreek on body weight, body composition, glucose tolerance, or insulin tolerance in the absence of high-fat diet (Figs 1–3). Furthermore, fenugreek supplementation did not affect food intake in either LF- or HF-fed animals (Fig. 1d). As expected, body weight and composition were substantially increased in mice fed HF diets compared to mice fed LF (Fig. 1a–c). However, there were no detectable differences in body weight or composition between HF and HFFG groups (Fig. 1a–c). Oral glucose tolerance testing revealed that fenugreek supplementation to HF-fed mice reduced glucose levels at selected times following glucose administration at 5, 11, and 15 weeks (Fig. 2) although the total area under the curve (AUC) was not substantially affected. Finally, fenugreek had no effect on insulin tolerance at any time point in either HF- or LF-fed mice (Fig. 3).

### Fenugreek supplementation modulates LDL and HDL balance without affecting total cholesterol, triglycerides, glycerol, or non-esterified free fatty acids

**Figure 4:**
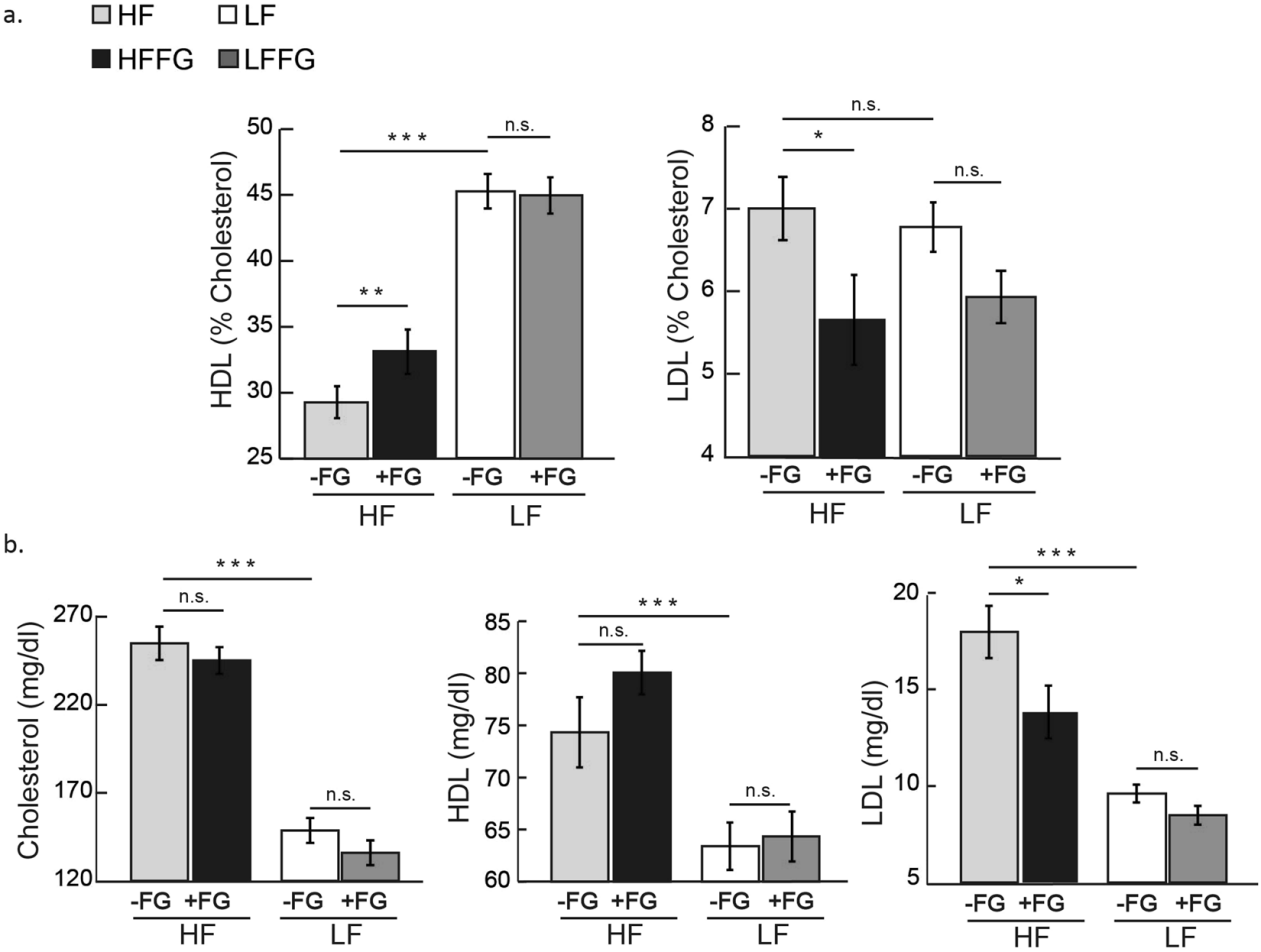
Fenugreek supplementation increased serum high-density lipoprotein (HDL) and decreased low-density lipoprotein (LDL) levels after 16 weeks on HF diet. Whole blood was collected, and serum was separated and analyzed for HDL, LDL, and total cholesterol following 16 weeks on LF, HF, LFFG, or HFFG diet. (**a**) HDL and LDL are represented as percent cholesterol and as (**b**) serum totals of HDL, LDL, and cholesterol, respectively. Results are presented as means ± SEM, (*n* = *12*). Comparisons were made by a 1-way ANOVA followed by student t-test. *p < 0.05, **p < 0.01, ***P < 0.001 was considered significant.

**Figure 5:**
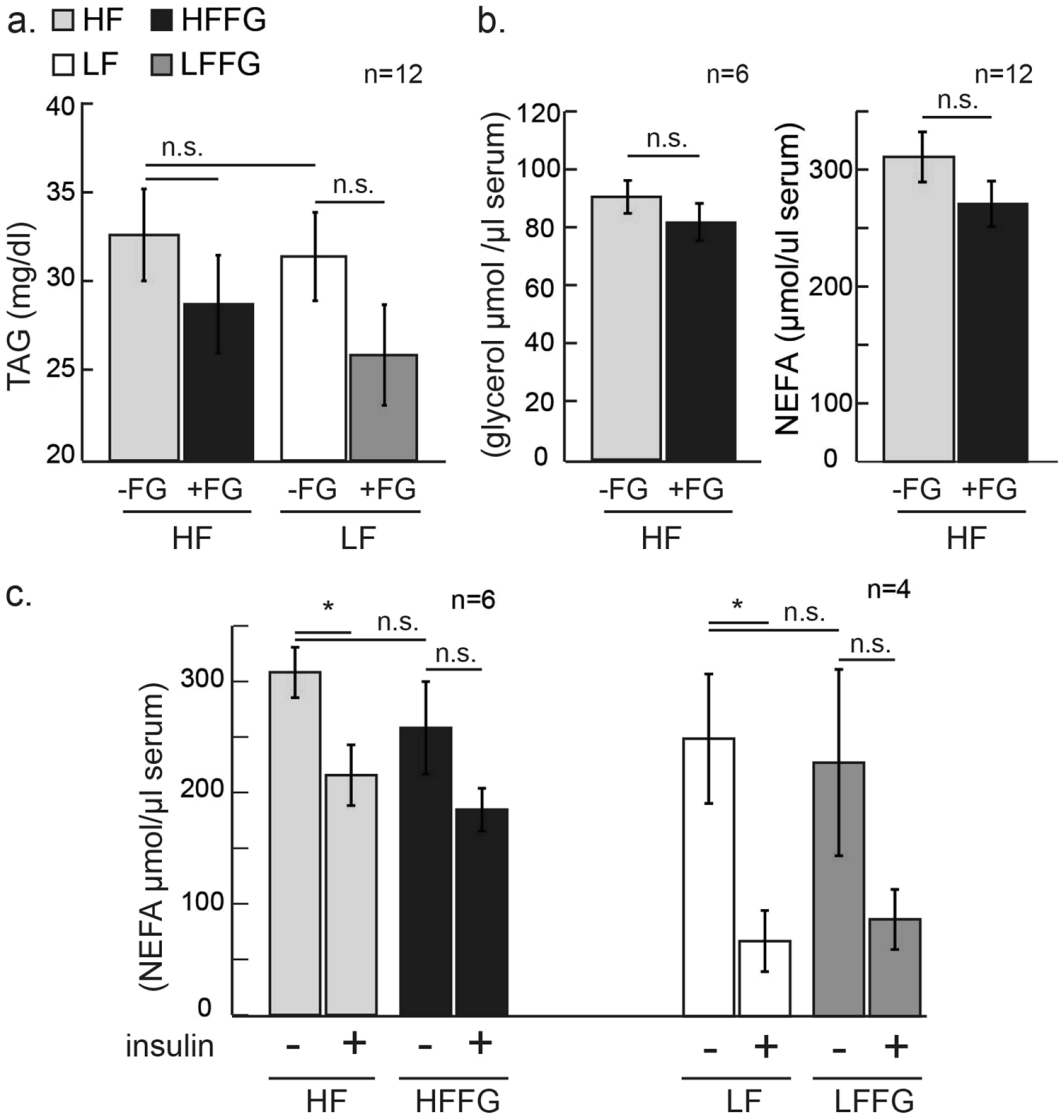
Fenugreek supplementation did not alter triglycerides (TAGs), glycerol, or non-esterified free fatty acids (NEFAs) in LF- or HF-fed mice. After 16 weeks on LF, HF, LFFG, or HFFG diet, half the animals were injected with insulin for 10 minutes and serum was analyzed for glycerol, TAGs, and NEFAs. (**a**) Total serum TAGs, (**b**) total serum glycerol and non-esterified free fatty acids, or (**c**) insulin-stimulated NEFAs. Data are presented as means ± SEM (*n* = *20 & 12*) as indicated. Comparisons were made by a 1-way ANOVA followed by student t-test. *p < 0.05 was considered significant.

Male C57BL/6J mice fed HF-diet exhibited increased total cholesterol compared to LF- fed mice, but total circulating cholesterol levels were unaffected by fenugreek supplementation in both LF- and HF-fed mice (Fig. 4b). Likewise, levels of both low-density lipoproteins (LDL) and high-density lipoproteins (HDL) were increased by HF-, and fenugreek supplementation decreased circulating LDL in HF-fed mice (Fig. 4b). Additionally, fenugreek supplementation increased HDL and decreased LDL, expressed as percent total cholesterol in HF-fed mice (Fig. 4a). There was no notable difference in HDL levels with fenugreek supplementation to LF-fed mice, and fenugreek did not affect circulating triglycerides, glycerol, or non-esterified free fatty acid (NEFA) when supplemented to either diet (Fig. 5a–c). As expected, acute insulin treatment reduced circulating NEFA in both HF- and LF-fed mice however, this effect was unaltered by fenugreek supplementation to either diet (Fig. 5c). These *in vivo* observations are consistent with several *ex-vivo* and *in vitro* studies showing that fenugreek does not affect basal or stimulated lipolysis in cultured murine adipocytes or subcutaneous or visceral adipose tissue (data not shown).

### Fenugreek supplementation increases adiponectin expression in subcutaneous inguinal adipose tissue, but does not alter HF-diet-induced inflammation in visceral epididymal adipose tissue

**Figure 6:**
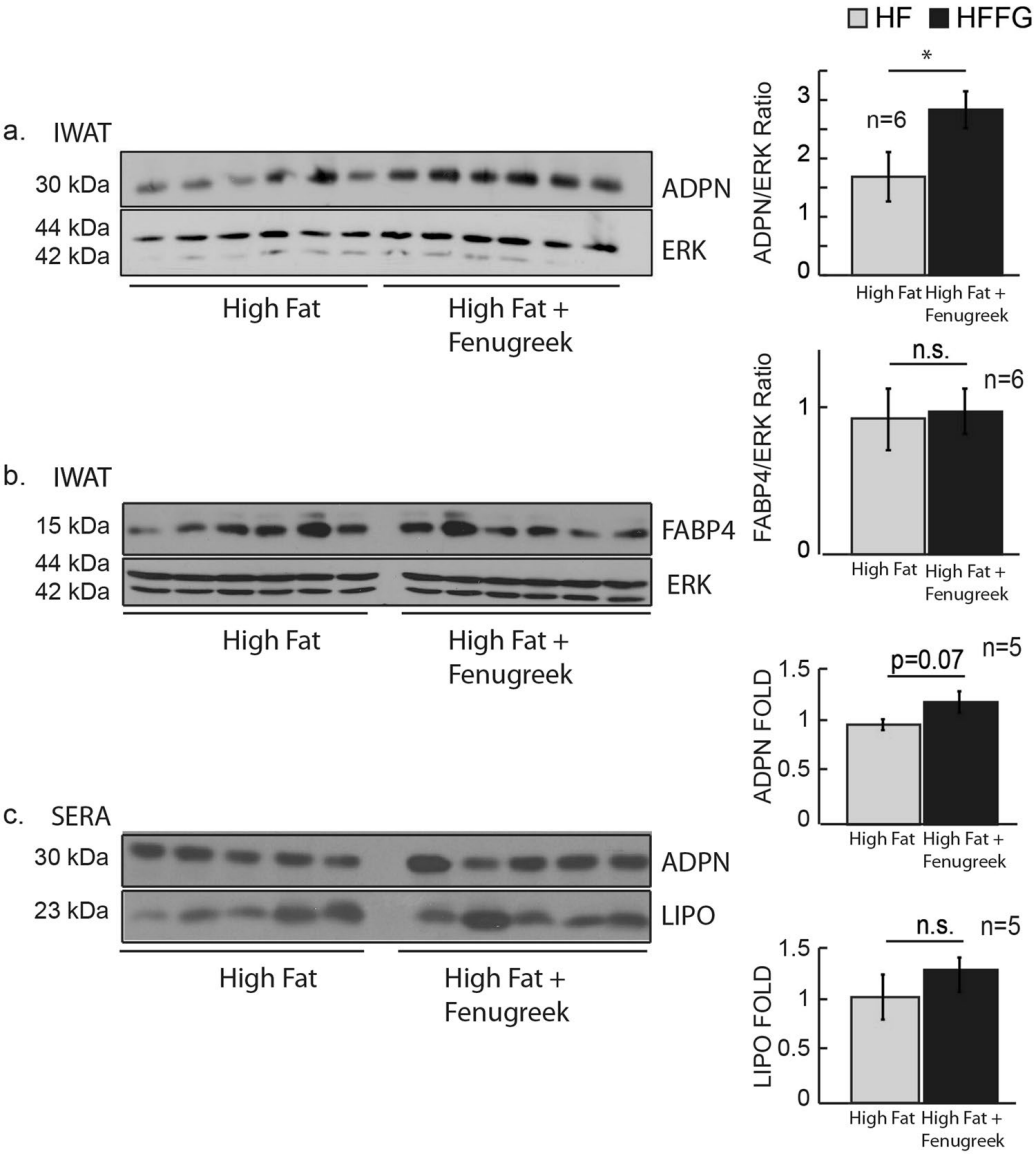
Fenugreek supplementation increased adiponectin expression, but not FABP4, in iWAT and modestly increased adiponectin in sera of HF-fed mice. Western blot analysis was performed on inguinal white adipose tissue (iWAT) and sera following 16 weeks on HF or HFFG diets. Western blot analysis was performed to examine adiponectin, FABP4, and lipocalin-2 (LIPO) expression. Each lane represents an iWAT tissue extract or sera aliquot from an individual animal. Cropped-representative blots of adiponectin^59^, FABP4^60^, LIPO^61^, and extracellular signal–regulated kinases (ERKs) 1 and 2^62^ are shown. iWAT adiponectin and FABP4 expression was normalized to ERK 1 levels while sera adiponectin and LIPO were normalized to total sera protein concentrations. Fold changes was determined by dividing each optical density measurement by the average density of the HF diet group. Results are presented as means ± SEM, (*n* = *6 and n* = *5*). Comparisons were made by a student t-test. *p < 0.05 was considered significant.

**Figure 7:**
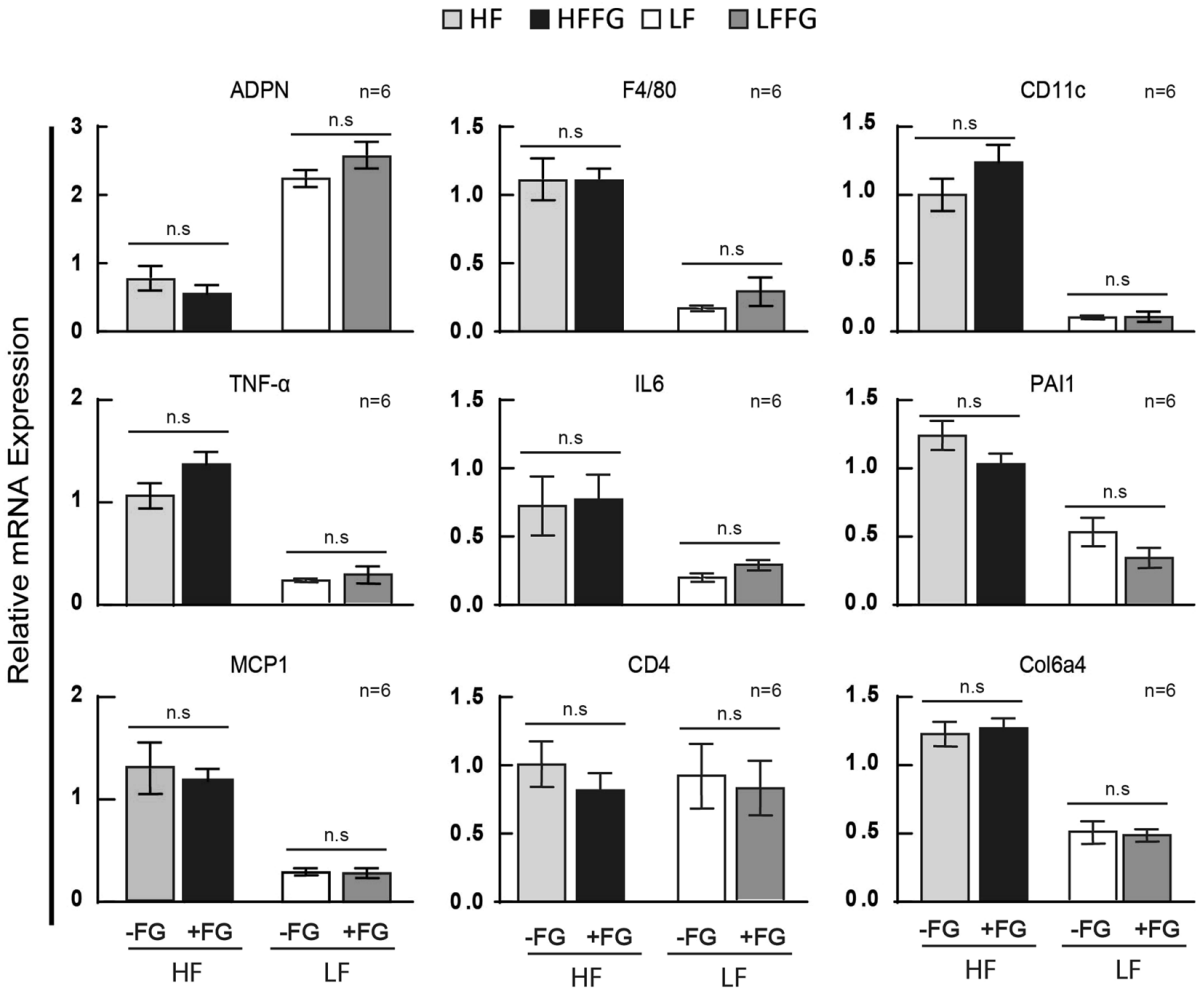
Fenugreek supplementation did not have anti-inflammatory properties in epididymal white adipose tissue. Relative mRNA abundance was assessed in eWAT from mice fed LF, LFFG, HF, and HFFG diets for 16 weeks to measure *adpn f4/80*, *cd11c*, *tnf-α*, *il-*6, *pai1*, *mcp-1*, *cd4*, and *col6a3*. Data were normalized to *cyclophilin a*. Results are presented as means ± SEM, *n* = *6*. Comparisons were made by a 1-way ANOVA followed by student t-test. *P < 0.05 was considered significant.

HF-feeding is known to reduce adiponectin expression in adipose tissue as well as in circulation^30^. Hence, the effects of fenugreek on diet-induced changes in adiponectin expression were examined in sera, subcutaneous, and visceral adipose tissue. As shown in Fig. 6a, fenugreek supplementation to HF-fed mice increased total adiponectin protein expression in subcutaneous inguinal adipose tissue (iWAT) when compared to HF diet alone. However, fenugreek did not affect HF-induced loss of adiponectin mRNA in visceral epididymal adipose tissue (eWAT) (Fig. 7). Fatty acid-bind protein 4 (FABP4/aP2) is a lipid binding protein that is a therapeutic target for metabolic disorders, and genetic deficiencies of FABP4 can improve glucose homeostasis^31^. Unlike adiponectin expression, FABP4 protein expression was not regulated in iWAT of HF fed mice with fenugreek supplementation (Fig. 6b). We also observed a modest, but not statistically significant (p = 0.07) increase in total circulating adiponectin levels with fenugreek administration in HF-fed mice (Fig. 6c). For comparison, we examined lipocalin-2 levels in circulation as well. This factor was unaffected by fenugreek administration (Fig. 6c).

There is some evidence of that fenugreek has anti-inflammatory effects^18–21^, and HF-feeding is known to induce inflammation in eWAT. Hence, the gene expression of the pro-inflammatory markers *f4/80, cd11c, tnf-α, il-6, pai1, mcp1, and col6apha3* was assessed in eWAT. As shown in Fig. 7, HF-feeding increased expression of all these inflammatory markers, but fenugreek supplementation did not alter this expression in eWAT of HF- or LF-fed mice.

### Fenugreek supplementation does not increase insulin-positive area or circulating serum insulin levels

**Figure 8:**
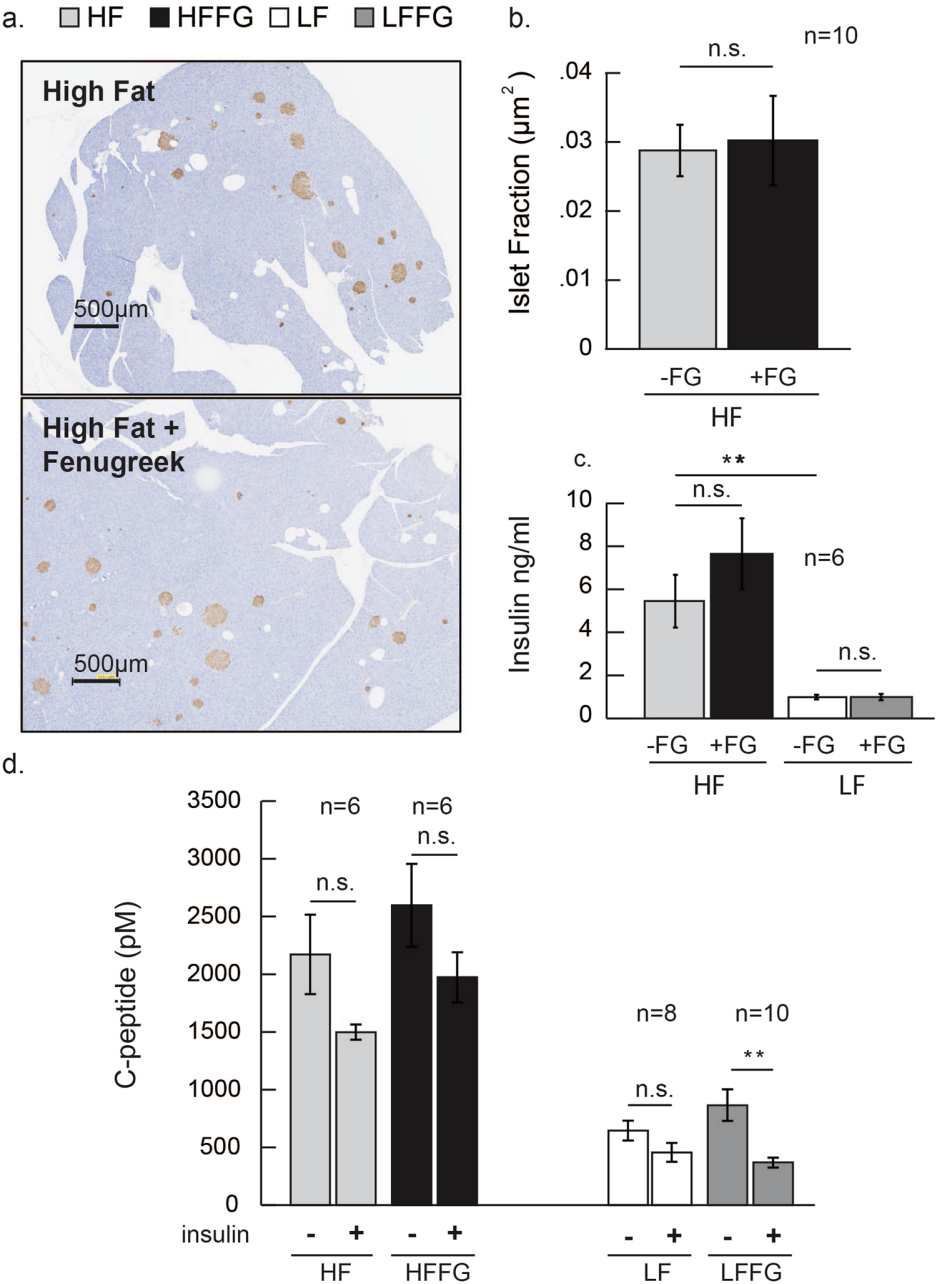
Fenugreek supplementation did not increase beta cell mass or circulating insulin and C-peptide levels. Histology sections were performed on the pancreas of mice exposed to HF or HFFG diets for 16 weeks. βeta cells were stained for insulin and counter stained with hematoxylin and eosin (H&E). In mice injected with saline or insulin^63^ for 10 minutes, serum was collected for insulin and C-peptide analyses via ELISA assay (**a**) Representative micrographs (10x) of insulin positive (brown) beta-islet cells. (**b**) Quantification of insulin-positive beta cell-islet to tissue fraction (**c**) Insulin, and (**d**) C-peptide ± insulin injection (1 U/kg). Data are represented as means ± SEM, (*n* = *6*, *8* *, &10*), as indicated. Comparisons were made by a 1-way ANOVA followed by student t-test. *p < 0.05 **p < 0.01, ***P < 0.001 was considered significant. Scale bar = 10 microns.

Fenugreek supplementation modestly improved glucose tolerance (Fig. 2), but not insulin sensitivity (Fig. 3). Since fenugreek has previously been reported to possesses insulinotropic bioactivity^5–8^, it was important to determine if the observed glucose tolerance effects of fenugreek were the result of altered insulin secretion during HF-feeding. As anticipated, HF-fed mice displayed increases in circulating insulin and C-peptide levels (Fig. 8c,d), but these increases were unaltered by fenugreek supplementation. Also, fenugreek did not affect pancreatic insulin positive β-cell area (Fig. 8a,b). Data show that while C-peptide levels were generally higher in HF-fed mice, fenugreek supplementation did not affect C-peptide responses to insulin in the context of HF-diet.

### Fenugreek supplementation decreases liver FABP4/aP2 expression, but does not affect FAS expression or triglyceride accumulation

**Figure 9:**
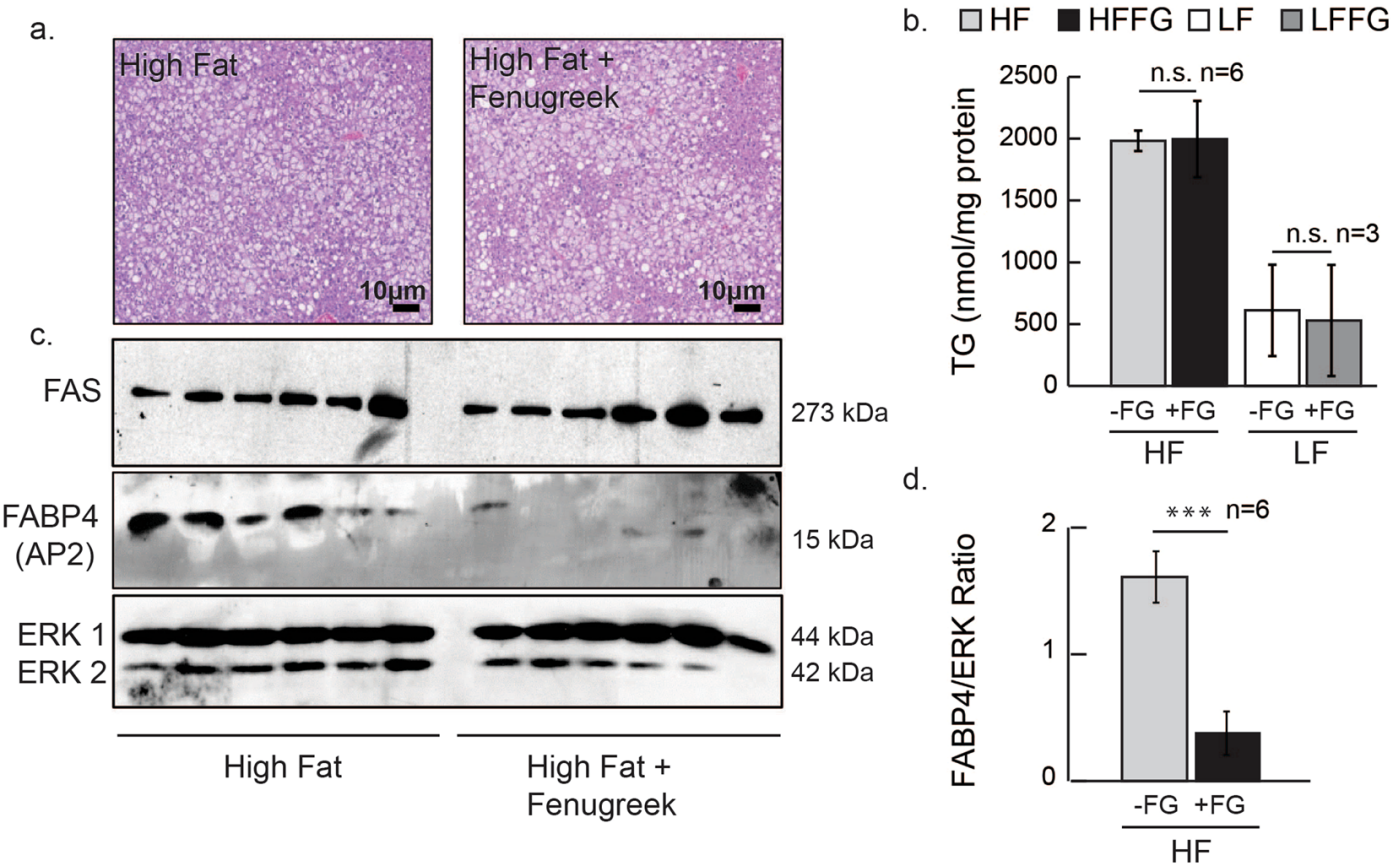
Fenugreek supplementation did not affect hepatic lipid accumulation, but substantially decreased liver FABP4/aP2/expression in HF-fed mice. The livers of mice exposed to HF or HFFG diets for 16 weeks were fixed and H&E stained, or collected for Western blot and tissue triglyceride analysis. (**a**) Representative photomicrographs of livers stained with H&E. (**b**) Quantification of liver tissue triglyceride content. (**c**) Western Blots of liver tissue extracts. The expression of fatty acid synthase (FAS)^64^, /fatty acid-binding protein 4 (FABP4/aP2), and extracellular signal–regulated kinases (ERK 1 and 2)^62^ is shown. Each lane represents a liver tissue extract from an individual animal. (**d**) FABP4 quantification normalized to ERK 1. Data are ﻿presented as mean ± SEM, (*n* = *6*). Comparisons were made by a 1-way ANOVA and/or by student t-test. *p < 0.05 **p < 0.01, ***P < 0.001 was considered significant. Scale bar = 10 microns.

Some evidence exists that fenugreek protects against hepatic steatosis^14–18^, while other evidence supports the contrary^29^. Since fenugreek supplementation altered the LDL and HDL ratio, but did not alter total cholesterol levels, it was important to determine if whole fenugreek seed supplementation prevented hepatic lipid accumulation and synthesis. As expected, there was an increase in liver triglycerides (Fig. 9a,b) in HF-fed compared to LF-fed mice. However, fenugreek supplementation did not attenuate HF-induced liver triglyceride accumulation or reduce fatty acid synthase (FAS) protein levels (Fig. 9b,c). Interestingly, fenugreek did dramatically reduce the expression of fatty acid-binding protein 4 (FABP4/aP2) when compared to HF-diet alone (Fig. 9c,d) relative to ERKs 1 and 2 expression.

### Fenugreek supplementation preserves fat mass, but was not as effective as voluntary wheel running in improving body weight or glucose tolerance in HF-fed mice

**Figure 10:**
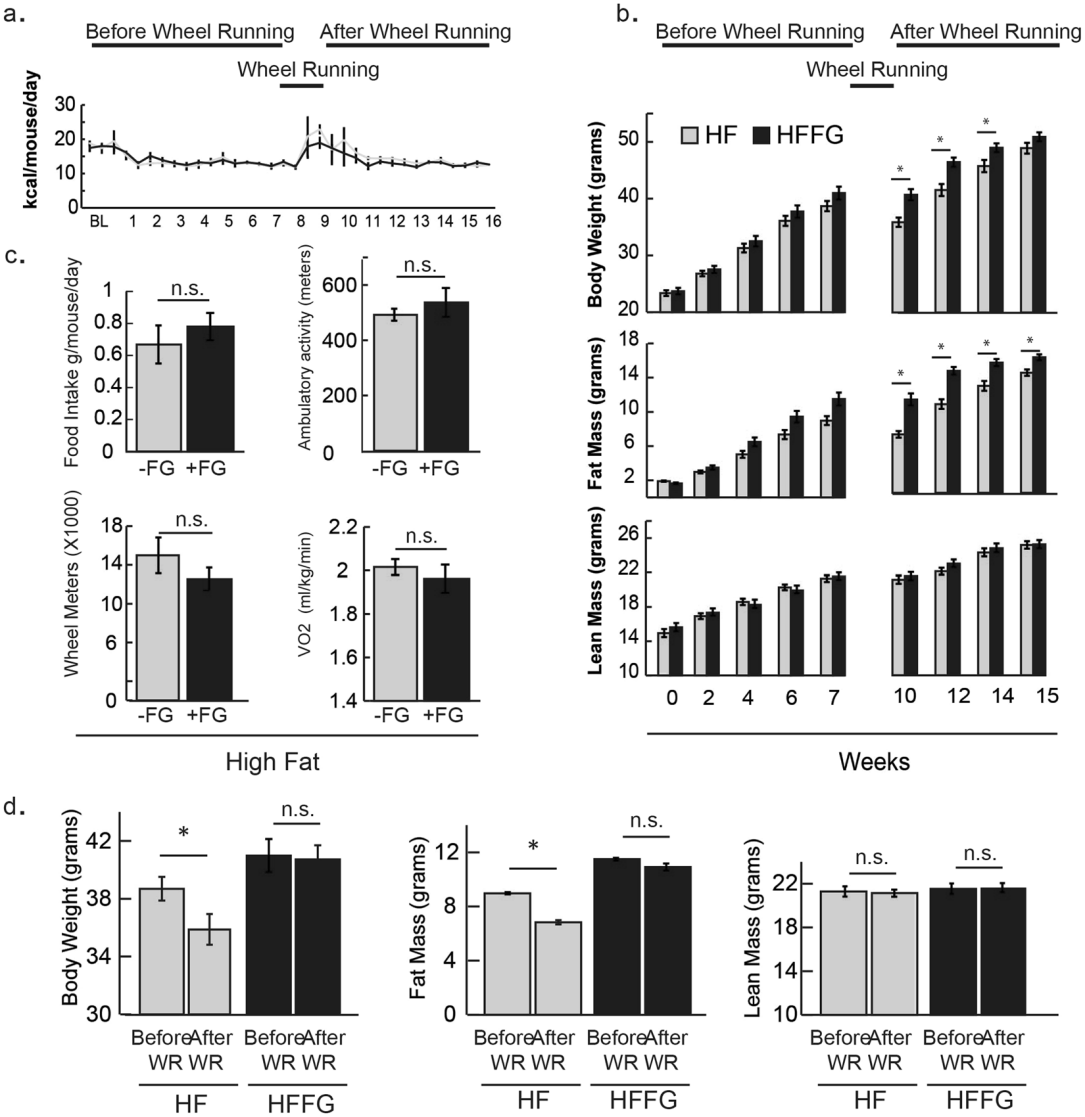
Fenugreek supplementation preserved fat mass in HF-fed mice exposed to voluntary wheel running (WR). Sixteen mice were separated from the original cohort, placed in single housed cages, and given access to voluntary wheel running for 4 days. (**a**) Caloric food intake was measured twice a week (**b**,**d**), while body weight, fat mass, and lean mass, were measured every two weeks before and after exposure to voluntary wheel running during 16 weeks of HF ± FG-feeding. (**c**) Food intake, ambulatory activity, WR, and volume of oxygen utilization were also measured during the 4-day exposure to voluntary WR. Results are presented mean ± SEM (*n* = *8*). Comparisons were made by a 1-way ANOVA followed by student t-test. *p < 0.05 was considered significant.

**Figure 11:**
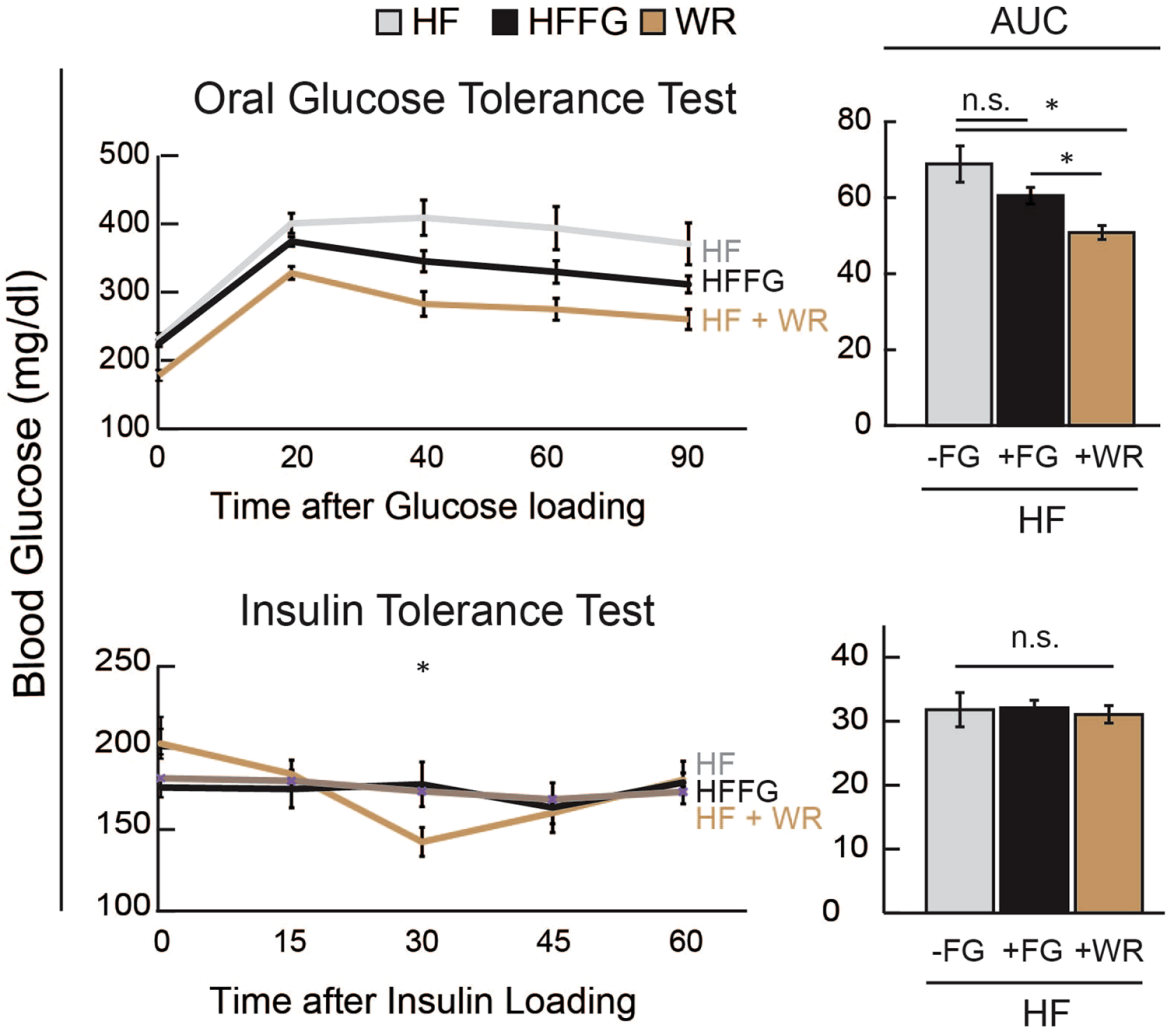
Fenugreek supplementation was not as effective as voluntary wheel running in improving glucose tolerance in HF-fed mice. OGTT was performed 2 weeks and ITT was performed 3 weeks after mice were exposed to WR for 4 days. AUC of OGTT after 2 weeks and ITT after 3 weeks of mice exposed to WR. Results are presented as means ± SEM, (*n* = *8*). Comparisons were made by a 1-way ANOVA followed by student t-test. *p < 0.05 was considered significant.

Since calorie restriction and exercise have been shown to impact insulin-stimulated glucose uptake^32^ and metabolic health^33^, we compared fenugreek supplementation to the effects of voluntary calorie restriction and wheel running (WR). HF- and HFFG mice experienced no measurable differences in calorie intake before exposure to WR (Fig. 10a). As expected, animals with free access to WR had increased activity. However, only HF-mice lost a substantial amount of weight following WR. When comparing body composition measurements before and after WR, weight loss was specific to fat mass (Fig. 10b,d) despite no differences in food intake, ambulatory activity, oxygen consumption, or total distance traveled during four days of WR (Fig. 10c). When glucose and insulin tolerances were measured in mice given HF-diet, fenugreek supplementation (HFFG), or temporary access to voluntary running wheels (HF + WR), data revealed that fenugreek was not as effective at improving glucose tolerance as WR alone (Fig. 11).

## Discussion

In our studies, we used C57BL/6J mice to examine the effects of dietary fenugreek supplementation to both LF- and HF-diets. The C57BL/6J mice are commonly used in obesity and Type 2 diabetes research as the males readily gain weight and develop insulin resistance with high-fat feeding^27^. When compared to LF-fed controls, HF-fed mice without fenugreek exhibited elevated body weight throughout the study. Supplementation with whole fenugreek seed (2% w/w) incorporated into food by Research Diets (New Brunswick, NJ) did not affect body weight, body composition, caloric intake, or insulin resistance for either LF- or HF-fed mice. However, transient improvements in glucose tolerance were documented, as were improvements in HDL to LDL ratios. In addition, we observed elevated adiponectin expression in iWAT and substantially decreased FABP4/aP2 levels in the liver of mice supplemented with fenugreek for 16 weeks on a HF-diet. Furthermore, fenugreek administration was not as effective in improving glucose tolerance in HF-fed mice as four days of wheel running. Based on an extensive literature of the anti-diabetic effects of fenugreek administration, [reviewed in^1^], we expected more improvements in metabolic health than observed in the current study.

Several studies support hypolipidemic effects of fenugreek in normal and diabetic subjects^26,34–45^; but we did not observe any substantial effects of fenugreek on circulating NEFAs, glycerol, or TAGs (Fig. 5). Nonetheless, we did examine the effects of fenugreek on lipolysis *in vitro* using 3T3-L1 adipocytes and *ex-vivo* in both subcutaneous and visceral mouse adipose tissue. Consistent with our *in vivo* observations, fenugreek did not have any effects on basal or stimulated lipolysis in adipocytes or adipose tissue (data not shown). These results are corroborated by a recent study by another laboratory that used a large range of fenugreek doses and did not observe any effect of fenugreek on lipolysis^46^. Although we expected more profound effects of fenugreek supplementation with HF-feeding, our results do suggest that fenugreek can improve a few markers of cardiovascular health in the context of high-fat feeding. Our results clearly indicate that fenugreek increases adiponectin levels in subcutaneous iWAT and there is modest, but not statistically significant, increase in adiponectin in circulation (Fig. 6). Increased adiponectin is strongly associated with cardiovascular health^47^, but adiponectin expression does not always correlate with insulin sensitivity in mice and humans^48–51^. In addition to increasing adiponectin expression in subcutaneous adipose tissue, fenugreek supplementation increased the percentage of HDL and decreased LDL in total cholesterol during HF-feeding (Fig. 4). One of the most striking observations of fenugreek administration was a decrease in aP2/FABP4 levels in the liver of HF-fed mice (Fig. 9). FABP4 is a lipid binding protein that is a therapeutic target for metabolic disorders, and genetic deficiencies of FABP4 have been shown to improve glucose homeostasis^30^. A small molecule FABP4 inhibitor has been shown to attenuate atherosclerotic lesion formation and improve endothelial function in ApoE^−/−^ mice^52,53^. Loss of FABP4 in macrophages has also been shown to protect ApoE^−/−^ mice against atherosclerosis^54^. While, FABP4 has also been shown to effect atherosclerotic lesion formation in severe hypercholesterolemia^55^. Collectively, these observations suggest that fenugreek may promote cardiovascular health in animals fed a HF-diet by decreasing aP2 expression in liver. Additional studies will be needed to determine if FABP4 expression in liver by fenugreek is due to inhibited expression in hepatocytes or macrophages.

There are many published studies on the ability of fenugreek to lower glucose levels [reviewed in ^1^]. In our studies, both five week and eleven week fenugreek supplementations resulted in modest improvements in glucose tolerance in HF-fed mice compared to LF-controls (Fig. 2). It should be noted these effects occurred in the absence of changes in food intake or alterations in body mass. One limitation to our study is that we only examined male mice and fenugreek may have improved, diminished or had similar effects in female mice. However, it is well known that female C57BL/6J mice are resistant to diet induced obesity^56^. Another limitation to our study is that we sacrificed the mice after the observed reduction in beneficial effects of fenugreek supplementation on glucose tolerance (Fig. 2). A few days prior to sacrifice there were little to no effects of a sixteen-week fenugreek supplementation on glucose tolerance (Fig. 2). We were also surprised that we did not observe any changes in insulin tolerance (Fig. 3) or adipose tissue inflammation (Fig. 7). To our knowledge, there are only two other studies that examine fenugreek supplementation to C57BL/6J mice fed a HF-diet and both studies are from the same research group. In the first study, mice were given a daily oral gavage of a hydro-alcoholic fenugreek extract (2 g/kg daily) for 18–20 weeks^28^. In these experiments, they observed decreased serum glucose, insulin and TAG levels, and less insulin resistance as calculated by HOMA-IR^29^. The second study used the same dose and delivery method for 20 weeks and liver lipid accumulation was assessed. Similar to our studies, these studies concluded that fenugreek had minimal or no effect on hepatic steatosis^29^. There are several explanations for why our glucose- and insulin-related measurements are distinct from these studies. First, we performed the more rigorous analysis of performing OGTTs and ITTs rather than examining fasting insulin and glucose. Also, HOMA-IR is a proxy for insulin resistance, but arguably a sub-optimal measure, particularly in mice^57^. Finally, the mice in our study were only fed a HF-diet for 16 weeks as opposed to 18 and 20 weeks in the other studies^28,29^. Any one or all of these factors could account for the differences in our observations regarding the amplitude of the anti-diabetic effects of fenugreek in high fat-fed C57BL/6J male mice.﻿ Another consideration in the interpretation of our results is that we incorporated whole fenugreek seeds into the diet. Some of the health-promoting effects of fenugreek have been attributed to identified-phytochemical-bioa﻿ctives (reviewed in^1^) and these bioactives may have different bioavailabilities within each delivery method.

Although we observed changes in glucose tolerance with fenugreek at some treatment times (Fig. 2), we did not observe changes in insulin tolerance at these times (Fig. 3). The lack of an effect of fenugreek on insulin tolerance (Fig. 3) is supported by our data indicating that fenugreek supplementation did not affect insulin levels (Fig. 8). Since there are a variety of other hormones that can regulate glucose levels, these observations are not entirely unexpected.

In our studies, we did not observe any effects of fenugreek administration on insulin secretion (Fig. 8) or the ability to confer anti-inflammatory properties in visceral adipose tissue (Fig. 7). There is evidence that one of fenugreek’s phytochemical bioactives, 4-hydroxyisoleucine, can increase insulin secretion^5,58^, however this has not been observed in mice fed whole fenugreek seeds^28^. Treatment of KK-Ay mice with a high fat diet supplemented with 2% fenugreek has been shown to reduce macrophage infiltration in adipose tissue^23^. Although high-fat diet increased the expression of several macrophage and pro-inflammatory markers in visceral adipose tissue including *f4/80, tnf, cd11c, il-6* and *pai1* (Fig. 7), we did not observe reduced adipose tissue inflammation with fenugreek administration in our study. It is possible that a HF-diet treatment greater than 16 weeks is needed to observe anti-inflammatory effects of fenugreek in adipose tissue or that the effects of a 60% HF diet were too extreme for whole seed fenugreek supplementation to continuously mitigate.

It is well known that calorie restriction and exercise have an impact on insulin-stimulated glucose uptake^32^ and metabolic health^33^. However, no study to date has performed a side by side comparison of fenugreek supplementation and exercise. In our studies, HF- and HFFG-mice exposed to WR (wheel running) resulted in reduced food intake as compared to HF- sedentary animals. There were no detectable differences in food intake, ambulatory activity, wheel running distance, or oxygen consumption between HF- and HFFG during four days of WR, but there was a reduction in body weight of HF-fed mice. This reduction was specific to fat mass. Glucose and insulin tolerance tests on HF-, HFFG, and HF + WR mice revealed that 16 weeks of fenugreek was not as effective at improving glucose tolerance as four days of WR. These results suggest that weight loss is more effective at improving glucose tolerance than fenugreek supplementation in this model system. It is not clear why HFFG mice retained fat mass after the exercise intervention, but it is known that fenugreek promotes adipocyte development^23^, and we have confirmed that fenugreek can promote adipocyte development *in vitro* (data not shown).

In summary, we completed a comprehensive study where 80 male mice were fed low- or high-fat diet in the presence or absence of fenugreek supplementation. These studies were conducted in a controlled and rigorous manner. Our observations were not as compelling as other studies, which demonstrate robust anti-diabetic effects of fenugreek supplementation. However, our study did produce results consistant with current literature^28^, despite the fact that the time and delivery of fenugreek administration were different. Based on our results, future studies should examine additional parameters of cardiovascular health following fenugreek supplementation. Also, extended fenugreek treatment times or reductions in diet fat composition might enhance the ability of this botanical to further promote metabolic resiliency. Nonetheless, these studies reveal novel, but modest effects of fenugreek supplementation, and we are hopeful that the inclusion of some of these negative observations may help investigators design improved studies to assess the efficacy of fenugreek administration to promote metabolic or cardiovascular health.

## Materials and Methods

### Seed Identification and Verification

*T. foenum-graecum L*. “Fenugreek” seeds were purchased from Johnny’s Selected Seeds (Winslow, Maine) and certified for organic sprouting. For *in vivo* studies, powdered Fenugreek seeds were prepared at Rutgers and delivered to Research Diets Inc., (New Brunswick, NJ). For incorporation into treatment diets, fenugreek seed powder comprised 2% of the diet by weight (w/w).

### Animals

Animal care and use was approved by the Institutional Animal Care and Use Committee at Pennington Biomedical Research Center (PBRC) and all experiments were in accordance with the National Institutes of Health Guide for the Care and Use of Laboratory Animals. Eighty male, C57BL/6J (6 weeks of age) were purchased from Jackson Laboratories and multi-housed in a filter top cage at (22 ± 2 °C) and humidity (50 to 60%) with a 12-h light/dark cycle for the duration of the experiment. Animals were given *ad lib* access to water and diets. Animal studies were conducted with 12 to 20 mice per group. All mice were maintained on chow diet prior to administration of experimental diets. Mice subjected to diet-induced obesity were fed a high-fat diet (HF) or HF plus 2% fenugreek seed powder (HFFG) consisting of 60% kcal from fat (Research Diets Inc.; D12492, D16020410 from 8 to 24 weeks of age). C57BL/6J control mice were also fed a low-fat diet (LF) consisting of 10% kcal from fat or a LF containing 2% fenugreek seed powder (LFFG) (Research Diet Inc.; D12450J, D16020408) from 8 to 24 weeks of age. The total time on experimental diets was 16 weeks. All diets contained 10% kcal from protein with the balance in caloric intake provided by differences in carbohydrate content. Food intake was measured biweekly. Body weight and body composition were measured every two weeks via digital scale (Saturus lab instruments, Göttingen Germany) and nuclear magnetic resonance (NMR) (mini spec LF110; Bruker, Billerica, Massachusetts, USA). All animals were injected with insulin 1.0 U/kg or saline (vehicle, equal volume) 10 minutes prior to anesthesia by CO_2_ gas asphyxiation and euthanasia via decapitation.

### Oral Glucose Tolerance Test (OGTT) and Insulin Tolerance Test (ITT)

All analyses were performed on conscious mice. For OGTT, a weight standardized glucose dose of 2 grams/ kg was administered via oral gavage after a 4 hour fast. Blood glucose was measured at 0, 20, 40, 60 and 90 minutes via a drop of blood drawn from the tail vein. For insulin tolerance test (ITT), an intraperitoneal injection of 0.5 U/kg insulin (Humulin-Regular 100U, Eli Lilly and Company Indianapolis, Indiana) was administered using a 23-gauge syringe. Blood glucose was measured at 0, 15, 30, 40, and 60 minutes via a drop of blood drawn from the tail vein. All blood glucose levels were measured using Breese 2 Glucose meter (Bayer, Parsippany, NJ).

### Serum Analysis

Whole blood was collected after decapitation and serum was separated by centrifugation at 3000 RPM in a Microtainer (Bectan, Dickinson and company Franklin Lakes, NJ) within 2 hours of collection. Levels of total non-esterified fatty acids, total cholesterol, HDL cholesterol, LDL cholesterol, and triglycerides in serum were measured colorimetrically (Wako Chemicals, Richmond, VA). Mouse insulin and C-peptide were evaluated by ELISA (Crystal Chem Inc., Downers Grove IL and American Laboratory Products Company, Salem, NH) and total serum adiponectin and lipocalin-2 expression were determined by western blot analysis.

### Tissue collection

Inguinal and epididymal adipose tissues were rapidly excised, flash frozen in liquid nitrogen, and stored at −80 °C for biochemical analysis. A portion of liver tissue was flash frozen in liquid nitrogen and stored at −80 °C for RNA, protein, and triglyceride biochemical analysis, while another portion was fixed in 10% neutral buffered formalin (NBF) for paraffin imbedding and histology. Pancreas tissue was also fixed in 10% NBF and paraffin imbedded for histological analysis.

### Pancreatic Beta Cell Mass Analysis

For assessment of β-cell mass, paraffin pancreatic sections were immunostained for insulin using a Guinea Pig anti-insulin antibody (Invitrogen). Briefly, pancreas samples were fixed in 10% NBF and processed on a Tissue-Tek VIP 6 Vacuum Infiltration Processor. Paraffin sections of 5 μm each were prepared. Two sections (separated by 150 µm from each pancreas were analyzed. Slides were scanned using a Hamamatsu Nanozoomer Digital Pathology (NDP) system (Hamamatsu City, Japan) at 20X. Digital images were analyzed with Visopharm. β-cell mass was calculated as the ratio of insulin positive β-cells area/total pancreas cross-sectional area.

### Protein Extract Preparation

Mouse tissues were homogenized in buffer containing 10 mM Tris (pH 7.4), 150 mM NaCl, 1% triton-X100, 0.5% Igepal, 1 mM Ethylenediaminetetraacetic acid (EDTA), 1 mM ethylene glycol-bis(β-aminoethyl ether)-N,N,N′,N′-tetraacetic acid (EGTA), (IP-Buffer) with protease as well as phosphatase inhibitors: 1 mM phenylmethylsulfonyl fluoride, 1 μM pepstatin, 2.5 µg/ul aprotinin, and 4.746 µg/ul leupeptin, 200 µM Sodium vanadate, and 100 µM Sodium Fluoride. For media samples collected from cell monolayers, 1 mM phenylmethylsulfonyl fluoride was used for protease inhibition. Protein content from cells, sera, media, adipocytes, and mouse tissue was determined using a bicinchoninic acid assay (Pierce Biotechnology, Rockford, IL, USA) according to the manufacturer’s instructions.

### Gel Electrophoresis and Immunoblotting

Tissue protein extracts were separated using polyacrylamide gels containing sodium dodecyl sulfate and transferred to nitrocellulose membrane (Bio-Rad) using 25 mmol/L Tris, 192 mmol/L glycine, and 20% methanol. Blots were blocked in 4% non-fat-dry-milk at room temperature for 1 hour and probed with appropriate primary antibody overnight at 4 °C. Proteins were visualized with horseradish peroxidase-conjugated secondary antibodies (Jackson ImmunoResearch Laboratory) and enhanced chemiluminescence (Pierce) using Premium Blue X-ray film (Phenix Research products, Candler, NC) and developed using mini-medical Series Developer, Mount Kisco, NY).

### qPCR

Total RNA from eWAT adipose tissue was isolated by column purification (QIAGEN) and yield was determined by spectrophotometry (NanoDrop Technologies). From each RNA sample, 200 ng was reverse transcribed to cDNA using the High Capacity cDNA Reverse Transcription kit (Applied Biosystems). All qPCR data collections were performed using an ABI PRISM 7900 (Applied Biosystems) platform. Relative expression for each target gene was calculated using a standard curve, and all target mRNA gene expression data were normalized to the reference gene, cyclophilin a. Mouse primers were purchased from Integrated DNA Technologies (Coralville, Iowa). Gene and primer sequences are as follows:

*m cyclophilin-a* fw: 5′-CCACTGTCGCTTTTCGCCGC-3′

re: 5′-TGCAAACAGCTCGAAGGAGACGC-3′

*m f4/80* fw: 5′-ATAGCTTCCGAGAGTGTTGTG-3′

re: 5′-TCCAACTGCTCTAACTCTGTG-3′

*m cd11c* fw: 5′-CTACCCGAGCCATCAATCAG-3′

re: 5′-GCTCTGCTTTCTACTGAGTTCA-3′

*m il-6* fe: 5′-TCCTCTCTGCAAGAGACTTCCATCC-3′

re: 5′-AAGCCTCCGACTTGTGAAGTGGT-3′

*m mcp-1* fw: 5′-GCAGAGAGCCAGACGGGAGGA-3′

re: 5′-TGGGGCGTTAACTGCATCTGG-3′

*m pai1* fw: 5′-CGGCAGAACCCGACAGAGACA-3′

re: 5′-TCCGAGGTCTGGGATGCTGGT-3′

*m adpn* fw: 5′-AAAAGGGCTCAGGACGCTACT-3′

re: 5′-TGGGCAGGATTAAGAGGAACA-3′

*m cd4* fw: 5′-CGTGATAGCTGTGCTCTGAA-3′

re: 5′-CTTCTCTCCATGTCCAACCTAA-3′

*m col6a3* fw: 5′-CCCTTCAGTGGTTGAAAGCG-3′

re: 5′-TGAGTCTGCGAACGATCCTG-3′

*m tnf-α* fw: 5′-AGACCCTCACACTCAGATCA-3′

re: 5′-TCTTTGAGATCC ATG CCGTTG-3′

### Statistical analysis

Data analyses were conducted using Microsoft Excel Spreadsheets and ABI PRISM 7900 (Applied Biosystems) and areas under the blood glucose response curves (AUC) were calculated for OGTT and ITT. All values were subjected to either a 1-way ANOVA or a two tailed student t-test. Results are expressed as mean ± SEM. A value of *P < 0.05, **P < 0.01, or ***P < 0.001 was considered significant.

### Data availability

All data generated or analyzed during this study are included in this published article.

## Electronic supplementary material

Supplementary Information
