# Supplementary material for: Fenugreek supplementation during high-fat feeding improves specific markers of metabolic health
Source: Sci Rep. 2017 Oct 6;7:12770. doi: 10.1038/s41598-017-12846-x (PMC5630574; doi:10.1038/s41598-017-12846-x)
Supplement: Supplementary file 1 — Supplementary Information [file 41598_2017_12846_MOESM1_ESM.doc]

**Fenugreek supplementation during high fat feeding improves specific markers of metabolic health**

Eric J, Knott^1^, Allison J. Richard^1^, Randall L. Mynatt^1^, David Ribnicky^2^, *Jacqueline M. Stephens^1^ and Annadora Bruce-Keller^1^

^1^Pennington Biomedical Research Center, Louisiana State University System, Baton Rouge, LA 70808.

^2^Department of Plant Biology, Rutgers University, New Brunswick, NJ 08901.

*Corresponding. jsteph1@lsu.edu

Supplemental Figure 1.


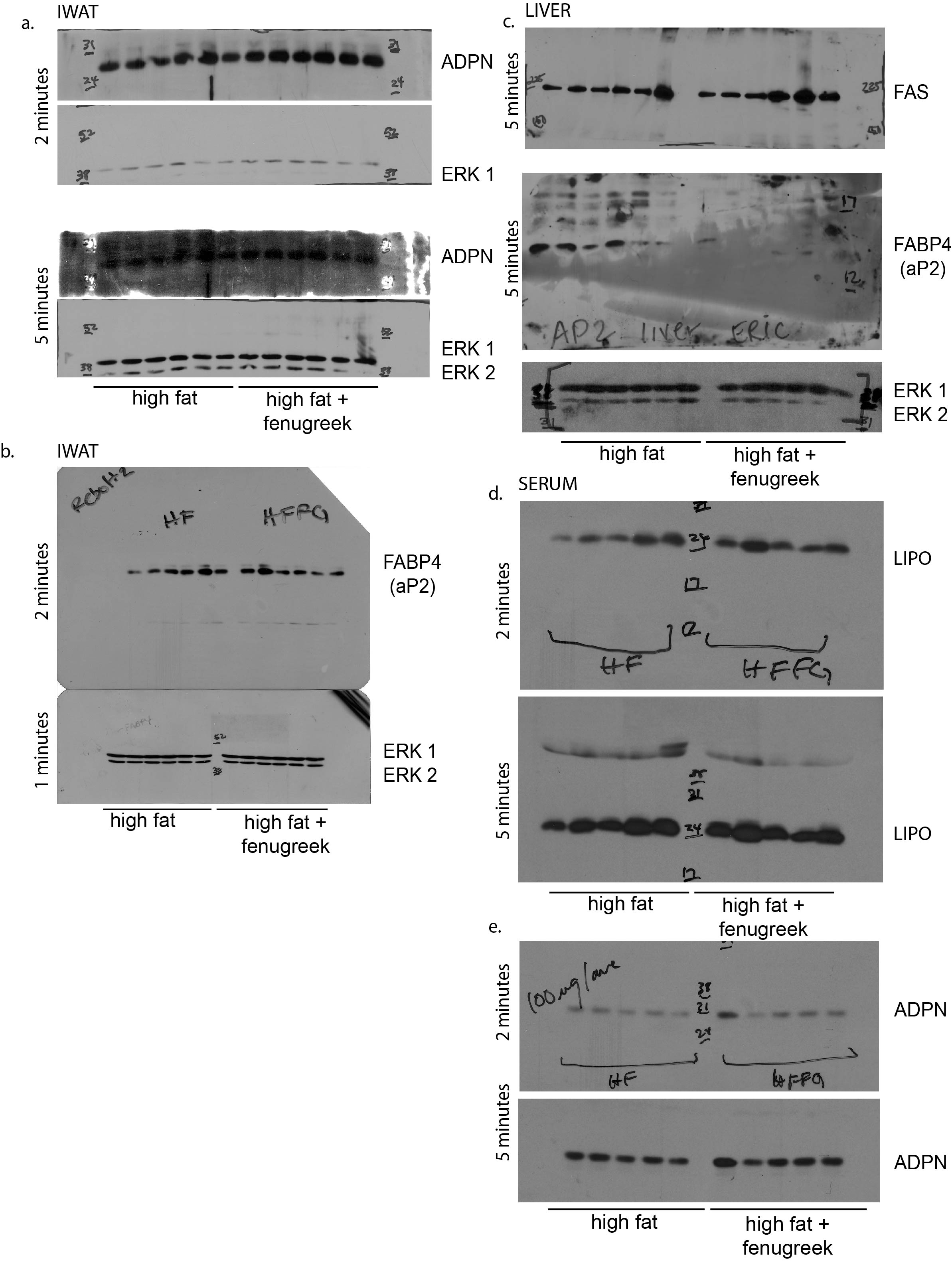


**Supplemental Figure 1.** **Original blot images of ADPN, FABP4, and LIPO (figure 6) and FAS and FABP4 (figure 9).** Membranes were physically separated based on protein of interest’s molecular weight (ERK, 42/44 kDa; FABP4, 15 kDa; FAS, 273 kDa; ADPN, 30 kDa, and LIPO, 23kDa) to probe separately. Post-probing, proteins were visualized with horseradish peroxidase-conjugated secondary antibodies (Jackson ImmunoResearch Laboratory) and enhanced chemiluminescence (Pierce) at various exposure times using Premium Blue X-ray film (size 5x7 or 8x10, Phenix Research products, Candler, NC), and developed using mini-medical Series Developer (Mount Kisco, NY). Original films were scanned using HP Scanjet N6310 in conjunction with Photoshop CS2 and protein bands quantified using Image Studio Lite version 3.1. These scanned images were cropped only to boarder the original cut of the physical membrane and auto-brightness/contrast applied using Adobe Photoshop CS5 Extended. The resulting Tiff images, resolution 300 dots per inch, were then exported into Adobe Illustrator CC and scaled accordingly. Displayed is a (a) two and five minute exposure of ADPN and ERK 1 and 2 for figure 6, (b) a two minute exposure for aP2/FABP4 and a one minute exposure of ERK 1 and 2 for figure 6 (c) a five minute exposure of ERK 1 and 2, FAS, and FABP4 for figure 9, and (d) a 2 and 5 minute exposure of ADPN and LIPO for figure 6.

Supplemental Figure 2.


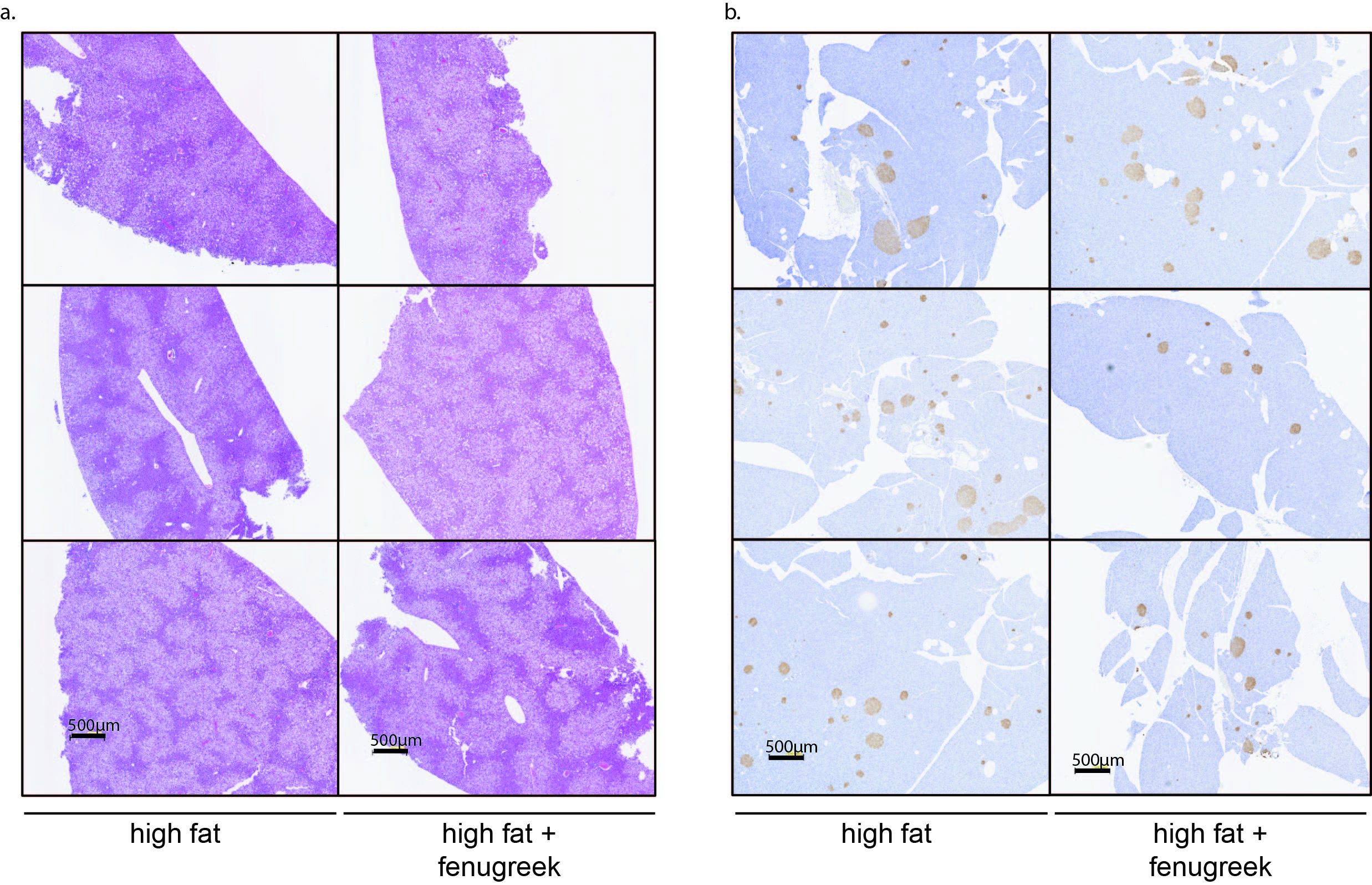


**Supplemental Figure 2. Sample micrograph images of pancreas (figure 8) and liver (figure 9).** Liver and pancreas samples were fixed in 10% NBF and processed on a Tissue-Tek 6 Vacuum Infiltration Processor overnight. (a) Liver paraffin sections of 5µm each were prepared. Two sections per sample were taken, and stained with Hematoxylin and Eosin (H&E) for morphological examination. The H&E was performed using a Lecia St 5020 Autostainer. Slides were scanned using a Hamamatsu Nanozoomer Digital Pathology (NDP) system scanner mode C9600-01 (Hamamatsu City, Japan, 20X-453nm/pixel, 49920x23808 pixels). (b) Pancreas paraffin sections of 5 μm each were prepared. Two sections separated by 150 µm were analyzed. Slides were scanned using a Hamamatsu Nanozoomer Digital Pathology (NDP) system scanner mode C9600-01 (Hamamatsu City, Japan, 20X-455nm/pixel). Digital images (53760x19968) pixels were analyzed with Visopharm. β-cell mass was calculated as the ratio of insulin positive β-cells area/total pancreas cross-sectional area.
